# Flattening the COVID-19 curve: Emotions mediate the effects of a persuasive message on preventive action

**DOI:** 10.3389/fpsyg.2022.1047241

**Published:** 2022-12-01

**Authors:** Krista Renee Muis, Gale M. Sinatra, Reinhard Pekrun, Panayiota Kendeou, Lucia Mason, Neil G. Jacobson, Wijnand Adriaan Pieter Van Tilburg, Ellen Orcutt, Sonia Zaccoletti, Kelsey M. Losenno

**Affiliations:** ^1^Department of Educational and Counselling Psychology, Faculty of Education, McGill University, Montreal, QC, Canada; ^2^Rossier School of Education, University of Southern California, Los Angeles, CA, United States; ^3^Department of Psychology, University of Essex, Colchester, United Kingdom; ^4^Department of Educational Psychology, College of Education and Human Development, University of Minnesota Twin Cities, St. Paul, MN, United States; ^5^Department of Developmental Psychology and Socialisation, University of Padua, Padua, Italy

**Keywords:** social persuasion, intervention, emotions, COVID-19, cross-cultural research

## Abstract

**Introduction:**

Across four countries (Canada, USA, UK, and Italy), we explored the effects of persuasive messages on intended and actual preventive actions related to COVID-19, and the role of emotions as a potential mechanism for explaining these effects.

**Methods:**

One thousand seventy-eight participants first reported their level of concern and emotions about COVID-19 and then received a positive persuasive text, negative persuasive text, or no text. After reading, participants reported their emotions about the pandemic and their willingness to take preventive action. One week following, the same participants reported the frequency with which they engaged in preventive action and behaviors that increased the risk of contracting COVID-19.

**Results:**

Results revealed that the positive persuasive text significantly increased individuals’ willingness to and actual engagement in preventive action and reduced risky behaviors 1 week following the intervention compared to the control condition. Moreover, significant differences were found between the positive persuasive text condition and negative persuasive text condition whereby individuals who read the positive text were more willing and actually engaged in more preventive action compared to those who read the negative text. No differences were found, however, at the 1-week follow-up for social distancing and isolation behaviors. Results also revealed that specific discrete emotions mediated relations between the effects of the texts and preventive action (both willing and actual).

**Discussion:**

This research highlights the power of educational interventions to prompt behavioral change and has implications for pandemic-related interventions, government policy on health promotion messages, and future research.

## Introduction

Since March 2020, COVID-19 has presented a serious threat to humanity and has forced individuals to assess the risk of their decisions daily. Two years into this global pandemic, it is critical that individuals continue to take preventive action to slow the rate of transmission and avoid overwhelming the medical system to save lives. Preventive action includes personal and social behaviors (e.g., washing hands more often, covering mouth when coughing or sneezing, wearing a mask), social distancing (e.g., remaining six feet apart from others), and isolation (e.g., not having visitors, staying inside if sick). Despite government mitigation strategies to slow the spread, Google tracking indicated that at the beginning of the pandemic, 41% of Canadians and 53% of Americans were still going to restaurants, cafes, shopping centers, theme parks, museums, libraries, and movies theatres, where social distancing would be practically impossible (Fournier, 2020; Google, 2020). Moreover, Abacus Data found that 25% of Canadians and 36% of Americans believed COVID-19 was not a serious issue (Abacus Data, 2020). Similar rates were reported in the UK (BBC, 2020). Given the seriousness of the pandemic, research in the behavioral social sciences (Pfattheicher et al., 2020) focused, in part, on identifying how best to persuasively communicate the personal and social risks of engaging or not engaging in preventive action.

To combat current global crises (e.g., climate change, anti-vaccination movements), research on social persuasion suggests that positive messages designed to increase perceived importance, relevance, and efficacy for engaging in social measures are more effective in changing individuals’ perceptions and increasing actions compared to neutral or negative messages (Muis et al., 2020; Thacker et al., 2021). Indeed, research has shown that persuasive messages are effective in changing perceptions and behavioral intentions (e.g., Jones et al., 2003; Berry et al., 2007). Moreover, recent research on COVID-19 suggests that public education health messages that focus on both public and personal benefits (“don’t get it, don’t spread it”) are more effective than addressing personal benefits alone (“don’t get it”) in increasing intentions to engage in personal and social preventive actions (Everett et al., 2020; Jordan et al., 2020).

Drawing on the broader social persuasion literature, although many studies have examined causal mechanisms with regard to changing perceptions and behavioral intentions, little research to date has examined one key factor in social persuasion–the role of emotions (Muis et al., 2020; Pfattheicher et al., 2020; Trevors and Kendeou, 2020; Thacker et al., 2021; Trevors, 2022). From a theoretical perspective, it is critical to understand what factors facilitate or constrain social persuasion. From a practical perspective, it is imperative to understand what kinds of persuasive messages prompt individuals to take action and save lives during a pandemic. This study aims to advance understanding of the role of emotions as a potential mechanism on the effects of persuasive messages in increasing both intended and actual preventive actions related to COVID-19 in the broader population. To do so, an immediate and 1-week delayed post-test experimental design was used in four different countries. Prior to delineating the specific research questions and hypotheses, we review relevant theoretical and empirical work.

### Social persuasion: The Elaboration Likelihood Model

Social influence through persuasion is one of the most prevalent civil means of social control available to governments and individuals (Briñol and Petty, 2009). Rather than forcing individuals or using threats to make them act in particular ways, persuasion provides an opportunity that is more likely to be successful, longer lasting, and beneficial for everyone. Broadly defined, persuasion includes influencing, convincing, or evoking a change in an individual’s understanding, beliefs, attitudes, behaviors or reactions toward a particular idea or premise (Murphy, 2001). The goal is to use reason and emotion to bring about change in another’s behaviors, understandings, or judgments of the topic under consideration (Murphy, 2001). Although many fields, like educational psychology, have examined what persuasion entails and how it unfolds, there is agreement across the literatures that characteristics of the learner (e.g., ability, relevance) and the message (e.g., source credibility, peripheral cues) play critical roles in persuasion (Petty and Cacioppo, 1986; Dole and Sinatra, 1998).

One of the most prominent models of persuasion is Petty and Cacioppo’s (1986) Elaboration Likelihood Model (ELM). According to Petty and Cacioppo, there are two routes to persuasion: the central route and the peripheral route. The central route requires effortful processing of the information pertaining to the object of focus. Two conditions are necessary for effortful processing to occur: the individual must be *motivated* and *able* to think deeply about the information. Motivation can be influenced by a number of factors including perceived relevance of the message, and whether the individual enjoys engaging in effortful thinking (i.e., need for cognition; Cacioppo et al., 1983). Ability is affected by the amount of distraction presented in the text, and the number of times the message is repeated (Cacioppo and Petty, 1979).

Persuasion through the peripheral route occurs when little effort is made to process the message, or very little attention is paid to it. The peripheral route is characterized by a reliance on simple cues (e.g., images and graphs) available in the persuasive message or context as well as heuristics (i.e., mental shortcuts) such as source evaluation. Although persuasion can occur through the peripheral route, the effects are typically short-lived (Haugtvedt and Petty, 1992). For longer lasting change to occur through persuasion, it is critical to engage individuals in more central route processing of the information. If individuals actively think about a message, the message is further elaborated on, and long-lasting change regarding the message is more likely (Petty et al., 2002). To promote more elaboration of messages, researchers have developed persuasive messages (Chambliss and Garner, 1996).

Persuasive messages are designed to challenge individuals’ beliefs and provide them with new information. In the context of COVID-19, a persuasive message may be a text that challenges individuals’ beliefs about the seriousness of the pandemic, and the importance of engaging in preventive action to protect oneself from getting it and for saving lives. Importantly, highly persuasive texts must be well written, provide sufficient evidence to support the arguments raised (Kendeou et al., 2014), come from credible sources like experts (Van Boekel et al., 2017), use powerful language (Areni, 2003; Blankenship and Holtgraves, 2005), and draw an emotional response from readers (Chambliss and Garner, 1996). In contrast to persuasive texts, expository or neutral texts include a description and explanation of a concept or topic that do not directly challenge individuals’ beliefs or behaviors, and do not include arguments to persuade individuals to change (Kendeou et al., 2019). For this research, we focused specifically on individuals’ emotional responses to assess how different emotions persuaded individuals to take preventive action.

### Emotions and persuasion

Emotions are recognized as critical to individuals’ attitudes, motivation, learning, and performance (Pekrun and Linnenbrink-Garcia, 2014; Sinatra and Seyranian, 2016). Emotion theorists define emotions as multifaceted phenomena that include affective, cognitive, motivational, physiological, and expressive components (Scherer and Moors, 2019). For example, anxiety that an individual has about the current pandemic situation may consist of feelings of uneasiness (affective), worry about getting COVID-19 (cognitive), desire to avoid people (motivation), increased heart rate and sweaty palms (physiological), and nervous facial expression (expressive; Pekrun and Stephens, 2012). Moreover, the type of emotion that arises can be described according to arousal (activating versus deactivating), valence (positive versus negative), and object focus (e.g., social emotions, topic emotions, achievement emotions).

Research has shown that positive emotional experiences, like happiness and hope, may increase effortful processing of information (Pekrun et al., 2009; Muis et al., 2015), whereas negative emotional experiences, like frustration and anger, often reduce effortful processing (Muis et al., 2015), as negative emotions draw attentional resources away from the task at hand (Meinhardt and Pekrun, 2003). For reading processes specifically, according to Bohn-Gettler’s (2019) Process-Emotion-Task (PET) framework, the influence that emotions have on reading comprehension will vary as a function of the nature of the task and the emotion being examined. For example, text-based research has shown that readers who experience higher positive emotions may engage in more assimilative processing, like backward inferences and elaboration, to integrate new information into existing mental representations compared to individuals who experience lower levels of positive emotions or more neutral or negative emotions (Bohn-Gettler and McCrudden, 2022). However, individuals who experience higher positive emotions may also ignore information that is inconsistent with their beliefs, which decreases the likelihood of changing those beliefs when their beliefs are challenged (Trevors and Kendeou, 2020). This suggests that positive emotions do not always result in improved processing of information and that context needs to be taken into consideration.

Additionally, when information is inconsistent with beliefs, this can trigger threat appraisals that prompt intense negative emotions, like anxiety, anger, and fear (Gregoire, 2003). When this occurs, individuals may be more likely to ignore belief-inconsistent information to protect their beliefs and avoid negative emotions, which may result in individuals learning less from those texts or changing fewer misconceptions, if at all, particularly about controversial topics (Trevors, 2022). However, negative emotions can also prompt accommodative processing (Bohn-Gettler, 2019). For example, information that is inconsistent with beliefs may initially trigger surprise (a neutral emotion), followed by confusion, frustration, or anxiety (Muis et al., 2018). These negative emotions, when they are not too intense, can signal to an individual that something is not quite right (Muis et al., 2018). When this occurs, individuals may engage in accommodation of existing knowledge or belief structures so that new information can be incorporated. Accordingly, as Bohn-Gettler (2019) has argued, context matters with regard to whether emotions will facilitate or constrain processing of information and subsequent belief and behavioral change.

In the COVID-19 pandemic context, researchers report that around the globe, as of 21 April 2020, individuals were experiencing negative emotions like anger due to lockdowns and removal of freedoms, sadness about the number of people who have died, fear about contracting COVID-19, a distrust in governments, and doubts about the seriousness of the pandemic (Xue et al., 2020). Individuals also expressed positive emotions including hope, joy, and empathy (Xue et al., 2020). These results suggest that individuals with misconceptions about the seriousness of the situation (Ecker et al., 2022), negative attitudes toward lockdowns, or negative emotions about COVID-19 may need to be persuaded to change their beliefs, understandings, emotions, or judgments so that they engage more deeply with the content (Murphy, 1998; Alexander et al., 2000) to increase the likelihood that they will take preventive action. It may also be the case the overly positive emotions that indicate a lack of understanding of the seriousness of the pandemic (like joy) may also need to be reduced so that individuals process the information more deeply to shift beliefs.

Accordingly, to persuade individuals to take prevention action to slow the spread of the virus and to save lives, it may be critical to develop a persuasive message that addresses both positive and negative emotions such that individuals are more likely to process the information and change beliefs about the seriousness of the pandemic. To date, research on the role of emotions in social persuasion has been limited (Thacker et al., 2021), but increasingly more research is exploring this issue in the context of the pandemic. For example, Heffner et al. (2021) focused on positive versus negative emotions and willingness to engage in social isolation *via* a threatening (e.g., millions will die) or pro-social text (e.g., “save millions of lives”). Results revealed that both texts were effective in increasing willingness to isolate, but that the threatening text was moderately unpleasant and highly arousing whereas the pro-social text was fairly pleasant and moderately arousing. In another study, Pfattheicher et al. (2020) found that empathy predicted willingness to engage in social distancing and wearing a face mask (Pfattheicher et al., 2020).

More research is necessary, however, to understand how specific discrete emotions may facilitate or constrain social persuasion. That is, it may be the case that emotions differentially predict individuals’ willingness to take preventive action. For example, although negative emotions often negatively predict learning from text, it may be the case that anxiety or sadness about COVID-19 increases individuals’ willingness to engage in, and actually take preventive action. Increased anxiety may drive individuals’ extrinsic motivation to process the content more deeply (Meinhardt and Pekrun, 2003) to ensure they do what they can to avoid getting COVID and saving lives. Fostering hope and empathy and decreasing anger and hopelessness may also be necessary to foster an increase in willingness to take preventive action. If individuals are angry about the lockdowns and do not believe the pandemic is a serious situation, they may need to be convinced that it is serious. An increase in perceptions of the seriousness of the pandemic may reduce their anger, thus allowing them to engage more deeply with the content. As Bohn-Gettler (2019) argued, context matters with regard to how emotions may facilitate or constrain text processing. That is, theoretically predictable patterns of relations between emotions and processing may vary as a function of the context in which the emotions occur. Accordingly, it is critical to examine how discrete emotions may facilitate or constrain the processing of persuasive messages in the context of the COVID-19 pandemic. Although some research in health promotion has demonstrated the effectiveness of persuasive messages and behavioral change through positively framed messages, to the best of our knowledge, none have focused on the role of discrete emotions on actual behavioral change.

### Elaboration Likelihood Model and health promotion

Since the early 1990s, health communication researchers have developed health promotion campaigns using the ELM and persuasive messages as a guide (Petty et al., 2009), targeting areas like exercise (Jones et al., 2003; Petty et al., 2017), AIDS and condom use (Carnaghi et al., 2007), smoking cessation (Flynn et al., 2011), and severe acute respiratory syndrome (SARS) education (Berry et al., 2007), among others. For example, to increase exercise intentions and behaviors, Jones et al. (2003) randomly assigned individuals to a positively or negatively framed communication from a credible or non-credible source. Results revealed that individuals who were given a positively framed message (benefits of exercise rather than a negative fear appeal) by an expert reported greater exercise intentions and actual exercise behaviors than individuals in the other conditions.

With regard to motivation, previous research on disease prevention, like vaccination decisions, has investigated self-interested versus pro-social motives to promote change in behaviors. The findings demonstrate that people have both self-interested and altruistic motives for vaccinations, and that targeting both types of motivations increases intentions to vaccinate (Hendrix et al., 2014; Li et al., 2016, Betsch et al., 2017). Of particular relevance, recent research on COVID-19 suggests that disease prevention messages that focus on both public and personal benefits are more effective in increasing preventive behavioral intentions compared to addressing personal benefits alone (Everett et al., 2020; Jordan et al., 2020). As such, messages should target both personal and public benefits while also invoking positive emotions, like empathy, and reducing negative emotions, like anger, to increase preventive actions. In contrast, more positive emotions like happiness may need to be reduced to ensure individuals do not ignore belief-inconsistent information.

## The current study

To date, although several studies have been conducted to examine the effectiveness of educational interventions on willingness to engage in preventive action during the COVID-19 pandemic (e.g., Everett et al., 2020; Jordan et al., 2020; Pfattheicher et al., 2020; Heffner et al., 2021), our understanding of the role of emotionally driven persuasive messages on increasing preventive action and reducing risky behaviors remains limited. Moreover, countries around the globe are not only at different phases of dealing with the pandemic, but also differ in their overall strategies and political systems. To examine the efficacy of different types of persuasive messages, we chose four countries that were at different phases at the onset of the pandemic and different phases of government action to slow the spread of the infection: Canada (lockdown in place for 3.5 weeks), USA (only some states beginning lockdown, such as California), UK (lockdown in place for 3.5 weeks), and Italy (lockdown in place for 6 weeks).

It is critical to better understand what persuasive messages are most effective in getting individuals to take preventive action (i.e., willingness and behavior), and whether emotions play a role in persuasion. The current research explores this pressing issue. The goal of this research was to develop a credible, powerful message on the seriousness of COVID-19, and to persuade individuals to take preventive action to stop the spread of the virus by focusing on personal relevance (i.e., don’t get it) and prosocial motives (gains focusing on saving lives). Across four different countries, individuals first reported their level of concern and general emotions about the pandemic, and then were randomly assigned to receive a negative message that focused on the number of deaths that could occur if individuals do not take preventive action, a positive message that focused on saving lives if preventive action is taken, or no message (control condition). Following this, participants in the two text conditions again reported their emotions about the pandemic, and then all participants rated their willingness to engage in preventive action and, 1 week later, reported actual preventive action taken.

Our research questions were as follows: (1) Are there differences in reported willingness to engage in preventive action as a function of condition (i.e., positive text condition, negative text condition, no text control condition)? (2) Are there differences in reported actual preventive actions as a function of condition 1 week following intervention? (3) Are there differences in reported emotions as a function of text condition? (4) Do emotions predict and mediate willingness to engage in prevention action, and actual preventive action 1 week following intervention?

Based on previous theoretical (Petty and Cacioppo, 1986; Briñol and Petty, 2009) and empirical work (Dryhurst et al., 2020; Jordan et al., 2020; Pfattheicher et al., 2020), we hypothesized that participants in the positive text condition would report a greater willingness to engage in preventive actions compared to the other two conditions, and that individuals in the negative text condition would be more willing to engage in preventive actions compared to the no text control condition (Hypothesis 1). We further hypothesized that participants in the positive text condition would report higher levels of actual preventive action, and lower levels of risky social behaviors compared to participants in the other two conditions, with participants in the negative text condition reporting higher levels of preventive action, and lower levels of risky social behaviors compared to participants in the control condition (Hypothesis 2). We also hypothesized that individuals in the positive text condition would report higher levels of positive emotions and lower levels of negative emotions compared to the other two conditions (Hypothesis 3), and that emotions would mediate relations between text condition and willingness and actual preventive action (Hypothesis 4). Based on previous research (Xue et al., 2020), we targeted the seven emotions that were most frequently reported around the globe concerning the current pandemic situation: happy, hopeful, empathetic, angry, anxious, sad, and hopeless.

**FIGURE 1:**
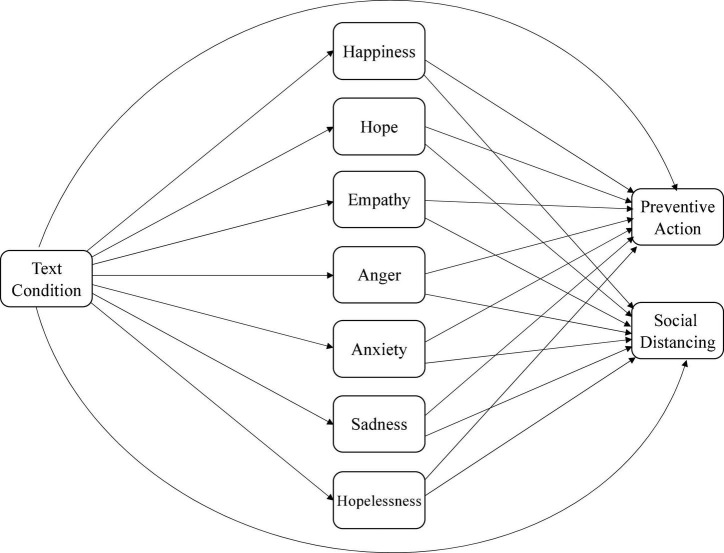
Hypothesized model of relations between text condition, emotions, and willingness to engage in preventive action.

**FIGURE 2:**
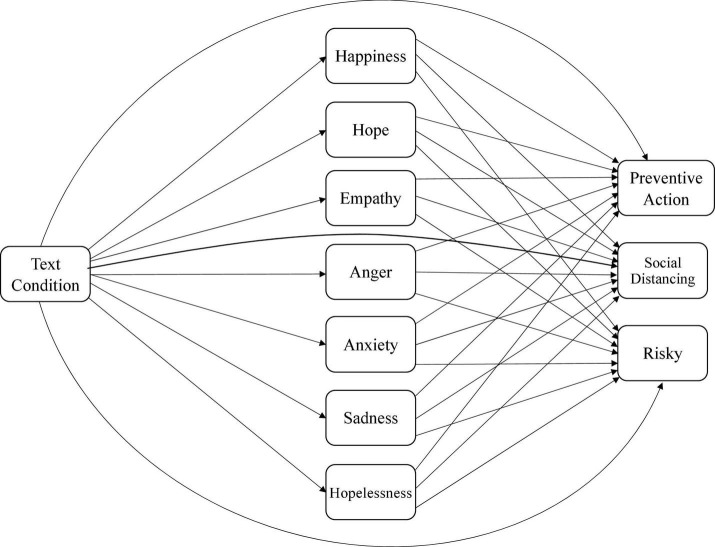
Hypothesized model of relations between text condition, emotions, and actual engagement in preventive action.

Specifically, we hypothesized that higher levels of happiness may reflect that individuals do not believe the pandemic situation is serious and will be less likely to engage in preventive action (Gregoire, 2003; Trevors and Kendeou, 2020; Trevors, 2022). Similarly, higher levels of anger may reflect individuals’ feelings about the restrictions and lockdowns and may be less likely to engage in preventive action. Higher levels of hopelessness may lead individuals to engage in less preventive action as they may perceive that preventive action will not help. In contrast, higher levels of hope, empathy, anxiety, and sadness about the pandemic may prompt individuals to take preventive action. See Figure 1 (for willingness) and Figure 2 (for actual preventive action) for the hypothesized models.

## Methodology

### Participants

We conducted a power analysis using G*Power (Version 3.1; Faul et al., 2007), which indicated that a sample of 432 would be necessary to detect a small effect of 0.15 (α = 0.05, power = 0.80). As such, we set a target of 450 participants for each country in case countries could not be merged and separate analyses by country were required.

#### Canada

One hundred seventy-five participants were recruited across Canada on 17 April 2020, using Amazon’s Mechanical Turk (MTurk). Another 327 participants from Canada were sampled by using a snowball sampling technique through Facebook. Of the 175 participants sampled using MTurk, three failed at least one of the two attention check questions and were subsequently removed, for a total of 172 participants from MTurk. Of the 327 participants sampled through Facebook, 292 completed the survey and passed both attention checks for a total sample of 464 (280 females, 26 did not respond) for the first survey, with 306 returning to complete the second survey (190 females). The average age was 39.84 years (*SD* = 14.49), with 83.1% reporting English as their first language, 40.1% reporting receiving a bachelor’s degree, and 50% reporting a personal annual income of $55,000 Canadian denomination (CAD) per year or less. For political views, 77% said they were liberal (Liberal, New Democratic, or Green), 15% said they were conservative, with the remaining being People’s Party or Bloc Québecois. With the exception of Prince Edward Island, Nunavut/Northwest Territories and the Yukon, all other provinces were represented.

#### USA

Four hundred seventy-six participants were recruited from across the USA on 17 April 2020, using Amazon’s Mechanical Turk (MTurk). An additional 95 USA MTurk participants were recruited on 20 April 2020. Of the 571 participants sampled, 183 were removed for spending less than 20 s reading the information or persuasive texts, 117 were removed for failing at least one attention check question, nine were removed for reporting non-USA zip codes, and eight were removed after being identified as duplicate participants. Of the remaining 254 participants, 173 completed the 1-week follow-up survey and passed both attention checks in the follow-up survey (*n* = 57 females). The average age of the sample was 35.91 years (*SD* = 11.13), with 89.4% reporting English as their first language, 59.4% reporting receiving a bachelor’s degree, and 51.2% reporting an annual income of $50,000 USD per year or less. For political views, 36% said they were conservative (Republican) and 64% said they were liberal (Democratic). Participants were grouped into four regions based on the first three digits of their zip code (16.1% Northeast, 36.6% South, 20.9% Midwest, 26.4% West).

#### UK

Four hundred fifty UK residents, representative of the population in terms of age, sex, and ethnicity (excluding those under 18 years of age), were recruited on 17 April 2020; 426 returned for the second part of the study. Participants were recruited through the crowdsourcing platform *Prolific.co*. Participants received a combined GBP £5.00 (USD $6.17) for taking part in both surveys. We excluded 21 participants who failed an attention check (*n* = 16), spent under 20 s reading the text (*n* = 4) or submitted an incorrect code (*n* = 1). Data for two returning participant IDs that could not be matched with the first survey were also dropped. The remaining sample (*N* = 429; 220 females, 207 males, 1 non-binary, 1 unreported) averaged 46.04 years in age (*SD* = 15.81). Of the participants, 53.4% possessed a university degree. Median household income was between GBP £35,000 and GBP £40,000 (USD $42,581 to USD $48,664). For political views, 18% reported being conservative, 48% reported being liberal, and 34% reported being neutral.

#### Italy

Two hundred forty-four participants across Italy were recruited from April 27th to April 29th, 2020, using Amazon’s Mechanical Turk (MTurk). Of these participants, three failed at least one of the two attention check questions and were subsequently removed. A total of 241 participants (158 females, 81 males, 1 non-binary sex, and 1 unreported sex) completed the first survey and 165 returned to complete the follow-up survey. The average age of the sample was 29.95 years (*SD* = 9.78), with 92% reporting Italian as their first language, 27% reporting a high school diploma, 13% a Bachelor’s degree and 24% a Master degree. For political views, 12% reported being conservative, 39% reported being neutral, and 49% reported being liberal. The reported median household income was between 15,000 and 20,000 € per year. Northern, Central, and Southern (with islands) Italy was represented.

### Materials

#### Experimental texts

Two experimental texts were adapted from Jordan et al. (2020), which were updated with the most current information on the day participants were recruited. With the exception of the emotional component, all features of the texts were identical [e.g., same credible sources were used (e.g., World Health Organization, Center for Disease Control and Prevention); written to be personally relevant; used persuasive language]. The first 269 words were identical across both texts, which began by providing participants with basic information about COVID-19, where it started, and the number of confirmed cases in Canada (or the USA, UK, or Italy, depending on the country in which participants were recruited) and world-wide. The text described the risk COVID-19 poses, the number of people in Canada (USA, UK, or Italy) that could be infected by the virus over the course of the pandemic, and the number of people who could die. It also stated how many people could require hospitalization, and how this high number could potentially crush the nation’s medical system due to a shortage of hospital beds, staff, intensive care units (ICUs) and ventilators. The text then stated how contagious COVID-19 is, described it as a serious threat, and recommended that the threat should be taken very seriously to prevent further spread.

The **negative text** then included a description about what each individual can do to keep safe from contracting (personal benefit) and spreading it (public benefit), including practicing good hygiene, engaging in social distancing, and self-isolating if even a bit sick. The text then ended with a graphic of the number of projected deaths over the course of the pandemic as a function of the percentage of the population being infected, which was taken directly from a technical briefing for Canadians (Public Health Agency of Canada, 2020; or other relevant country source). Total word count for the negative text was 342 (363 for the USA, 350 for the UK, and 483 for Italy), with a Flesch reading ease score of 41.1 (34.6 for the USA, 26.2 for the UK, and Gulpease index of readability was 57 for the Italian text where 100 = very easy), and a Flesch–Kincaid grade level of 11.7 (13.4 for the USA, and 16.8 for the UK).

The **positive text** included a description about what federal and provincial governments have done to stop the spread of the virus, and then stated that those actions are not enough; that we need to do more to stop the spread and save lives. The same preventive actions as in the negative text were then listed. The positive text next included a description of different scenarios in Canada (USA, UK, or Italy) if people take full action (i.e., all preventive actions), less action, or no action, and then asked people to do their part in saving lives by taking full action now. The same graphic as the negative text was then presented. Total word count for the positive text was 566 (580 for the USA, 537 for the UK, 731 for Italy), with a Flesch reading ease score of 41.1 (40.3 for the USA, 35.2 for the UK, Gulpease index = 56), and a Flesch–Kincaid grade level of 11.7 (12.5 for the USA, and 14.5 for the UK). See the Supplementary Appendix for each text used.

#### Text analysis

We conducted a textual analysis of the linguistic valence for each text to ensure the texts were valenced in the appropriate direction. That is, the positive persuasive text should be more linguistically valenced in a positive direction compared to the negative persuasive text (which should be more negative than the positive text). We used SEANCE 1.2.0 (sentiment analysis and social cognition engine; Crossley et al., 2017) using VADER. As a manipulation check, we also analyzed each text for positive emotions versus negative emotions. For valence, results revealed that the negative text had more negative valence (0.134) than the positive text (0.099) but that both texts were equivalent in positive valence (both at 0.06). For positive versus negative emotions, results revealed that the positive text used more positive emotions (0.08) compared to the negative text (0.06) and that the negative text used more negative emotions (0.07) than the positive text (0.06). In summary, these results suggest that with regard to the negative valence (both linguistic and emotional), the negative text was more negative than the positive text, and that the positive text was more emotionally positive than the negative text.

#### COVID-19 concern

A self-report questionnaire consisting of five items was used to measure participants’ concern about the pandemic. These items were taken from health-based research that assesses individuals’ perceived seriousness of an event; specifically, the negative consequences related to an anticipated health event in the future (e.g., getting COVID), or to a current pre-existing health problem (Rosenstock, 1974). As previous research over the past five decades has shown, concern predicts the likelihood that individuals will take action to prevent illness or disease (see Stretcher and Rosenstock, 1997). Example items included, “How concerned are you at present about the coronavirus pandemic?” and “In terms of the pandemic, how concerned are you about your own physical health?” Participants rated each item on a 5-point Likert scale with anchors for each value: 1 “Not at all,” 2 “A little,” “Moderately,” “Very much,” and 5 “Extremely” concerned. Cronbach’s alpha reliability estimate was good at α = 0.84.

#### Emotions

A self-report questionnaire consisting of seven items was used to measure participants’ emotions toward COVID-19. Each item consisted of a single word (e.g., “Happy”) and participants were asked to report the intensity of their emotional response to COVID-19 in relation to the pandemic prior to reading the text (all three groups) and again after they read the text (positive and negative text conditions). Research has shown that single-item measures are psychometrically sound substitutes for multi-item scales when administration time is limited (e.g., Gogol et al., 2014). Intensity was reported using a 5-point Likert scale with the following labels: 1 “Not at all,” 2 “Very little,” 3 “Moderate,” 4 “Strong,” and 5 “Very Strong.” Seven emotions were measured: happiness, hope, empathy, anger, anxiety, sadness, and hopelessness.

#### Willingness to engage in preventive action

A 22-item measure, adapted from Jordan et al. (2020), was used to assess participants’ willingness to engage in preventive action. Following recommendations provided by health authorities [e.g., center for disease control (CDC)], items were defined as (1) preventive actions to protect oneself (personal) and others (social) and, (2) social distancing and isolation. The first eight items measured participants’ willingness to engage in personal and social preventive actions using a sliding scale ranging from 1 (not at all willing to do this) to 100 (very willing to do this), with 50 (moderately willing to do this) in the middle. Examples included, “Wash my hands with soap for at least 15–20 seconds,” “Wipe down high-traffic surfaces at home with a disinfectant (e.g., door handles, counters, toilet levers, light switches),” and “Try my hardest to not touch my face.” The subsequent 14 items measured willingness to engage in social distancing and isolation actions using the same sliding scale. Examples included, “Keep at least 2 metres (6 feet) apart from people when I go outside for a walk or exercise,” “Limit trips outside for essential needs only (e.g., for getting groceries, medications),” “Stay home if I am not feeling well.” Cronbach’s alphas were high at α = 0.89 and 0.92 for the preventive and social distancing/isolation scales, respectively.

#### Follow-up preventive action behavior measure

In the 1-week follow-up survey, participants were given the exact same items as the willingness scale (but written in past tense, see below), with one dropped due to redundancy (i.e., “Stay at home, even if a bit sick” versus “Stay at home”). The first seven items measured individuals’ engagement in preventive action, and the next seven measured individuals’ engagement in social distancing and isolation. For those 14 items, participants were asked to rate the extent to which they engaged in the following actions over the past 7 days using a sliding scale from “0% of the time” to “100% of the time,” with “50% of the time” being the middle option. Compared to the original willingness scale items completed 1 week prior, items for the post-test were written in past tense rather than future tense. For example, the original item “Try my hardest not to touch my face” was rewritten as “Tried my hardest not to touch my face.” Other items included, “Washed my hands with soap before I ate,” and “Used an alcohol-based disinfectant if I did not have access to water and soap.” The remaining seven items required participants to report the number of times they actually engaged in specific behaviors over the past 7 days. These items were identical to those from the willingness scale, but because the sliding scale descriptors did not logically make sense with these items, participants were asked to provide the actual number with which they engaged in the behaviors (e.g., items like “have face-to-face gatherings with people who do not live with you” could not logically be reported on a sliding scale from 0% of the time to 100% of the time). Moreover, these seven items were behaviors that would put people at risk for contracting COVID, or spreading it to others, for example, “Use public transportation,” “Go shopping for non-essential goods,” and “Have visitors in your home who do not live with you.” As such, we labeled these items as “risky” behaviors, which were also summed across the seven items for a total “risk” score. Cronbach’s alpha for the preventive action and social distancing/isolation scales were good at α = 0.77 and α = 0.79, respectively. Given that the other scale was a frequency count of actual behavior, Cronbach alpha for reliability is not appropriate to compute (see Jones et al., 2003).

#### Demographic information

Participants reported their age, sex, first language spoken, highest level of education completed, political affiliation and views, and annual income.

### Procedure

Ethics was first approved by ethics boards at each respective university conducting the research in each country. After providing consent, participants were randomly assigned to one of three conditions: positive text, negative text, or no text (control condition). After providing consent, all participants completed the concern about COVID questionnaire followed by the emotions scale about COVID. Participants in the control condition then completed the willingness to engage in preventive action scale followed by the demographics questionnaire. For participants in the two text conditions, following completion of the emotions scale, they were presented with one of the texts and instructed to “Please read the information about COVID-19 carefully, which the World Health Organization has recently classified as a pandemic.” Once participants in the two text conditions read the texts, they were asked to report their emotions again and were then given the willingness to engage in preventive action scale followed by the demographics questionnaire. All participants were then asked to provide their Worker IDs (for MTurk) or emails (Facebook) if they were interested in participating in the 1-week follow-up survey.

Seven days after completion of the first survey, participants were invited to participate in the second survey. MTurk participants were paid $1 USD for each survey completed, *Prolific.co* participants were paid £4 for the first survey and £1 for the second survey, and participants sampled from Facebook were entered into a draw to win $100, with the chance of winning being 1 in 100, for each survey completed.

## Results

### Preliminary analyses

#### Data cleaning and screening

For outliers, 29 individuals reported frequencies of actual behaviors as unrealistic (scores ranging from 200 times to 2,000 times for items like “Wipe down high traffic surfaces at home”) and were deemed entry errors and were defined as missing data. For normality, as expected, all preventive actions were negatively skewed. Happy at pre-test and post-test was positively skewed (12.32 and 13.02). Hopeless at pre-test and post-test was also positively skewed (6.2 and 4.92) as was angry at post-test (5.95). Finally, empathy at pre-test was negatively skewed (–6.17).

To address the skewness issue, we used PROCESS for SPSS (Hayes, 2022) with bootstrap sampling which has no underlying distributional assumptions for mediation analysis (Hayes, 2022). PROCESS Model 4 with bootstrap sampling set to 10,000 and confidence intervals set at 95% were used to examine differences between groups on emotions and outcomes, to explore relations between emotions and outcomes, and to assess whether emotions mediated relations between text condition and outcomes. Specifically, with Model 4, indicator coding was used to examine mean differences between text conditions (entered as X variables) for both emotions (entered as mediators) and behavioral outcomes (entered as Y variables) with the control condition as the reference group (Hypothesis 1 and 2). Using Model 4 in PROCESS also allowed us to examine direct effects of emotions on outcomes (Hypothesis 3), and whether emotions mediated relations between text conditions and outcomes (Hypothesis 4).

Prior to conducting the analyses, we examined whether attrition for the 1-week follow-up was at random as a function of condition and demographic variables measured. Little’s test revealed that all missing data were missing completely at random (MCAR). For consistency purposes for analyses across the two time points, we then removed participants who did not complete both surveys (immediate and 1-week delay). Of the original 1,412 participants, 1,078 completed both surveys and were used for all analyses reported below. We then calculated intraclass correlation coefficients (ICCs) to assess whether nested analyses by country were needed given that each country was at a different stage of the pandemic and had different regulations in place with regard to government restrictions. For all outcomes, ICCs were less than 05. Multigroup analyses to assess measurement invariance (configural, metric, and scalar) across country samples for the outcomes of interest were then conducted. Results revealed that, with the exception of scalar invariance, configural and metric invariance held at each level for all outcomes. Given the low ICCs and that invariance held across samples, we combined all samples into one. We then assessed whether the willingness items would be better represented by a two-factor solution, as defined by the CDC, or as a one-factor solution. Results from the two-factor solution revealed a better fit of the model (CFI = 0.94, RMSEA = 0.07) than the one-factor model (CFI = 0.79, RMSEA = 0.12). Finally, no differences were found between groups on emotions prior to reading for happiness, *F*(2, 1077) = 2.79, *p* > 0.05, hope, *F*(2, 1077) = 1.41, *p* > 0.05, empathy, *F*(2, 1077) = 0.34, *p* > 0.05, anger, *F*(2, 1077) = 3.03, *p* = 0.05, anxiety, *F*(2, 1077) = 2.85, *p* > 0.05, sadness, *F*(2, 1077) = 3.08, *p* > 0.05, and hopelessness, *F*(2, 1077) = 1.80, *p* > 0.05.

#### Concern

To assess to what extent participants needed to be persuaded about the seriousness of the pandemic, we examined individuals’ level of concern. On average, participants were only moderately concerned about the pandemic, *M* = 3.22, *SD* = 0.89. Specifically, 36.2% of the sample was not at all to only a little concerned about the pandemic, 39.8% were moderately concerned about the pandemic, and 24% of the sample indicated they were very concerned to extremely concerned. We interpreted this as evidence that nearly half the sample needed to be persuaded about the seriousness of the pandemic and to take preventive action. We then assessed whether groups differed on level of concern about the pandemic. No statistical differences between groups were found, *F*(2, 1076) = 1.70, *p* = 0.18. Finally, to provide evidence that level of concern predicts the likelihood of taking preventive action, we computed correlations between concern and each of the preventive action outcomes. As expected, concern correlated with each of the preventive actions; personal preventive willingness *r* = 0.22, *p* < 0.001, social distancing willingness *r* = 0.18, *p* < 0.001, actual personal preventive action *r* = 0.25, *p* < 0.001, actual social distancing/isolation *r* = 0.08, *p* < 0.01, and risky behaviors *r* = –0.08, *p* = 0.04.

#### Treatment fidelity

To assess whether the text had the intended effect on participants’ emotions, we compared specific emotions that we expected would shift prior to and after reading the text based on the text content: happiness, hope, anger, sadness, and hopelessness (the control group did not receive a text, so emotions were measured only once for that group). We expected that individuals who read the positive text would likely remain consistent in their level of happiness (i.e., no decrease) or even a slight decrease given the nature of the topic, but not to the same extent as individuals who were given the negative text, who were expected to report a decrease in happiness. We also expected that individuals in the positive text condition would report a similar level of sadness and hopelessness (i.e., no decrease), but a decrease in anger and an increase in hope given the focus on saving lives. In contrast, we expected individuals in the negative text condition to report an increase in sadness and hopelessness, and a decrease in hope and anger given the focus on deaths.

Consistent with predictions, paired-samples *t*-tests revealed that individuals who were given the positive text reported no change in the level of happiness (*M* = 1.89, *SD* = 1.08; *M* = 1.80, *SD* = 0.98), *t*(346) = 1.49, *p* = 0.14, sadness (*M* = 3.12, *SD* = 1.22; *M* = 3.01, *SD* = 1.26), *t*(346) = 0.11, *p* = 0.91, or hopelessness (*M* = 2.37, *SD* = 1.21; *M* = 2.36, *SD* = 1.24), *t*(346) = 0.12, *p* = 0.90, after reading the text but did report a decrease in anger (*M* = 2.63, *SD* = 1.12; *M* = 2.38, *SD* = 1.20), *t*(346) = 3.58, *p* < 0.001, *d* = 0.20. In contrast to predictions, participants’ level of hope slightly decreased rather than increased (*M* = 3.05, *SD* = 1.00; *M* = 2.76, *SD* = 1.07), *t*(346) = 4.68, *p* < 0.001, *d* = 0.27. As expected, individuals who were given the negative text reported a decrease in happiness (*M* = 1.82, *SD* = 1.03; *M* = 1.63, *SD* = 0.90), *t*(323) = 3.80, *p* < 0.001, *d* = 0.21, hope (*M* = 2.94, *SD* = 0.97; *M* = 2.51, *SD* = 1.05), *t*(324) = 8.72, *p* < 0.001, *d* = 0.48, and anger (*M* = 2.63, *SD* = 1.11; *M* = 2.30, *SD* = 1.14), *t*(324) = 6.09, *p* < 0.001, *d* = 0.34, and an increase in hopelessness (*M* = 2.55, *SD* = 1.25; *M* = 2.65, *SD* = 1.25), *t*(336) = –1.99, *p* = 0.04, *d* = 0.11. No change in sadness (*M* = 3.19, *SD* = 1.23; *M* = 3.15, *SD* = 1.23), *t*(324) = 0.87, *p* = 0.38, occurred after reading the text. Except for sadness, we interpreted these results to suggest that the texts had the intended effect.

**FIGURE 3:**
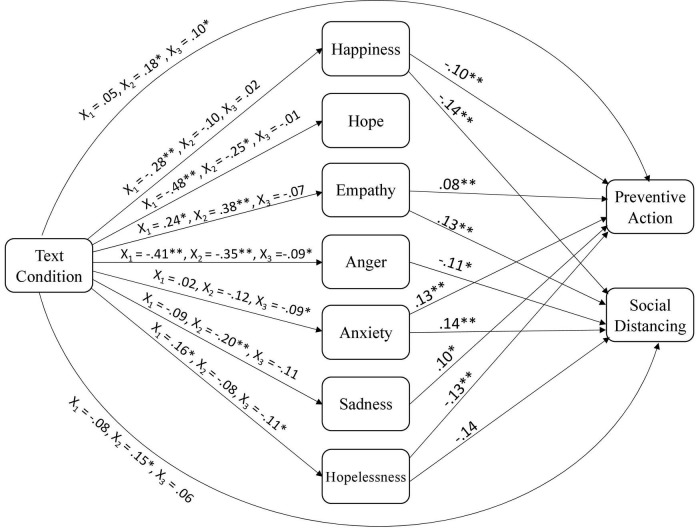
Model of relations between text condition, emotions, and willingness to engage in preventive action. Standardized effects; only significant paths are shown for emotions to outcomes to reduce complexity. *p < 0.05 and ^**^p < 0.01.

Means and SDs for all outcomes as a function of text condition are depicted in Table 1, and Table 2 reports the means and SDs of emotions for each text condition at pretest and posttest. Table 3 includes the zero-order correlations for all continuous variables. Figures 3 (willingness), 4 (actual) present the standardized direct effects of relations between text condition, emotions, and the various preventive outcomes.

**TABLE 1:** Preventive willingness and actual behavior as a function of text condition.

Outcome	Text	Mean	SD
Preventive willingness	Positive	92.81	12.00
	Negative	90.28	13.10
	No text	89.26	13.99
Social distancing willingness	Positive	87.54	10.56
	Negative	87.22	10.85
	No text	85.67	12.07
Preventive action	Positive	83.61	14.34
	Negative	79.27	16.58
	No text	81.53	16.52
Social distancing/Isolation	Positive	94.40	9.63
	Negative	94.59	8.38
	No text	93.25	11.88
Risky behavior	Positive	5.93	5.64
	Negative	7.54	6.84
	No text	7.72	6.31

Positive text condition, *N* = 347; negative text condition, *N* = 325; control condition *N* = 406.

**TABLE 2:** Emotions at pre- and post-test as a function of text condition.

Emotion	Text	Pre	Pre	Post	Post
				M	SD
Happiness	Positive	1.89	1.08	1.80	0.98
	Negative	1.82	1.03	1.63	0.90
	No text	1.98	1.12	1.98	1.12
Hope	Positive	3.05	1.00	2.76	1.07
	Negative	2.94	0.97	2.51	1.05
	No text	3.04	1.03	3.04	1.03
Empathy	Positive	3.31	1.15	3.06	1.22
	Negative	3.24	1.17	3.05	1.24
	No text	3.27	1.19	3.27	1.19
Anger	Positive	2.63	1.12	2.38	1.20
	Negative	2.62	1.11	2.30	1.14
	No text	2.80	1.20	2.80	1.20
Anxiety	Positive	3.18	1.19	3.08	1.22
	Negative	3.38	1.11	3.24	1.13
	No text	3.21	1.24	3.21	1.24
Sadness	Positive	3.12	1.22	3.00	1.26
	Negative	3.18	1.23	3.13	1.23
	No text	3.24	1.24	3.24	1.24
Hopelessness	Positive	2.37	1.21	2.36	1.24
	Negative	2.55	1.25	2.65	1.25
	No text	2.47	1.22	2.47	1.22

The control group completed the emotions questionnaire only once given that they were not presented a text. Pre and post for that group included the same data.

**TABLE 3:** Zero order correlations between variables.

		2	3	4	5	6	7	8	9	10	11	12
1	Willing Preventive	0.58**	0.60**	0.37**	−0.08*	−0.13**	–0.01	0.12*	–0.01	0.14**	0.11**	0.01
2	Willing Social		0.33**	0.49**	−0.24**	−0.16**	0.003	0.09**	−0.09**	0.09**	0.03	–0.05
3	Preventive			0.31**	–0.06	0.06*	0.07*	0.13**	0.07*	0.10**	0.07*	0.03
4	Social Distancing				−0.25**	−0.16**	–0.05	0.04	−0.08**	0.06	0.01	–0.03
5	Risky					0.11*	0.06*	0.04	0.01	0.004	–0.03	–0.01
6	Happy						0.44**	0.14**	−0.06*	−0.18**	−0.23**	−0.16**
7	Hope							0.16**	−0.09*	−0.19**	−0.17**	−0.30**
8	Empathy								0.11**	0.24**	0.26**	0.16**
9	Anger									0.35**	0.43**	0.40**
10	Anxiety										0.56**	0.57**
11	Sad											0.59**
12	Hopeless											

N = 1078. **p < 0.001, *p < 0.01.

**FIGURE 4:**
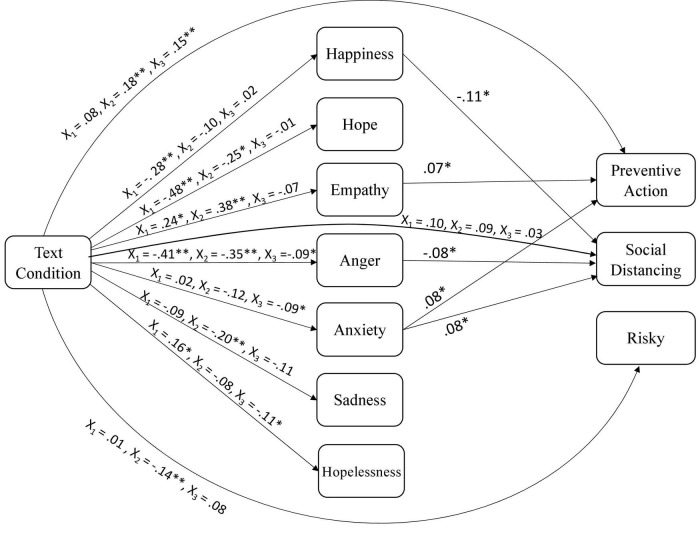
Model of relations between text condition, emotions, and actual engagement in preventive action. Standardized effects; only significant paths are shown for emotions to outcomes to reduce complexity. *p < 0.05 and **p < 0.01.

### Willingness to engage in preventive action

For the first research question, *whether text condition had an effect on willingness to engage in preventive action*, results revealed a significant main effect of text condition, *F*(9, 1067) = 6.58, *p* < 0.001. Specifically, no significant difference was found between the negative text condition and the control condition, *t* = 0.63, *p* = 0.54, but a significant difference was found between the positive text condition and the control condition, *t* = 2.45, *p* = 0.01, *d* = 0.27, and between the positive text condition and the negative text condition, *t* = 2.41, *p* = 0.02, *d* = 0.20. As hypothesized, the positive persuasive text significantly increased individuals’ willingness to engage in preventive action compared to the negative text condition and the control condition. Similarly, for willingness to engage in social distancing and isolation, results revealed a significant main effect of text condition, *F*(9, 1067) = 8.43, *p* < 0.001. Specifically, no significant difference was found between the negative text condition and the control condition, *t* = 1.03, *p* = 0.31, but a significant difference was found between the positive text condition and the control condition, *t* = 2.01, *p* = 0.045, *d* = 0.17. No difference was found between the positive text condition and negative text condition, *t* = 1.42, *p* > 0.05.

### Actual preventive action

For the second research question, *whether text condition had an effect on actual preventive action*, results revealed a significant omnibus test of the direct effect of text condition, *F*(9, 1073) = 7.54, *p* < 0.01. Results from the relative direct effects of text condition on engagement in preventive action revealed no significant difference between the negative text condition and the control condition, *t* = –1.09, *p* = 0.27, but a significant difference between the positive text condition and the control condition, *t* = 2.44, *p* = 0.01, *d* = 0.14, and between the positive text condition and negative text condition, *t* = 3.42, *p* < 0.001, *d* = 0.28. As hypothesized, the positive text significantly increased individuals’ engagement in preventive action 1 week following the intervention compared to the negative text condition and the control condition. In contrast, for engagement in social distancing and isolation, results revealed no significant differences between conditions for the seven items on the scale, all *p* > 0.05.

For engagement in risky behaviors (the other seven items for social distancing and isolation), results revealed a significant omnibus test of the direct effect of text condition on engagement in risky behaviors, *F*(9, 1051) = 4.64, *p* = 0.01. Results from the relative direct effects of text condition on engagement in risky behaviors revealed no significant difference between the negative text condition and the control condition, *t* = 0.20, *p* = 0.84, nor between the positive text condition and negative text condition, *t* = –1.76, *p* > 0.05. However, a significant difference was found between the positive text condition and the control condition, *t* = –2.49, *p* = 0.01, *d* = 0.32. As hypothesized, the positive persuasive text significantly decreased individuals’ engagement in risky behaviors 1 week following the intervention compared to the control condition. From a positive perspective, one can infer that the positive text increased individuals social distancing and isolation behaviors indirectly *via* a reduction in behaviors that put them at risk to getting or spreading COVID.

### Effect of text condition on emotions

For the third research question, *whether individuals’ emotional responses to the COVID-19 pandemic differed as a function of text condition*, results revealed a main effect of text condition on happiness, *F*(2, 1074) = 7.32, *p* < 0.001. Specifically, a significant difference was found in intensity of ***happiness*** between the negative text condition and the control condition, *t* = –3.81, *p* < 0.001, *d* = 0.39, but not between the positive text condition and the control condition, *t* = –1.46, *p* = 0.14 nor between the positive text condition and negative text condition, *t* = 0.54, *p* > 0.05. As can be seen in Table 2, the negative text significantly reduced individuals’ level of happiness about the pandemic situation as compared with the other two conditions (although not statistically different from the positive text condition), which was expected given the primarily negative tone of the message.

For ***hope***, results revealed a main effect of text condition, *F*(2, 1076) = 21.89, *p* < 0.001. Specifically, a significant difference in intensity of hope was found between the negative text condition and the control condition, *t* = –6.59, *p* < 0.001, *d* = 0.50, and between the positive text condition and the control condition, *t* = –3.51, *p* < 0.001, *d* = 0.27, but not between the positive text and negative text conditions, *t* = –0.17, *p* > 0.05. Participants in the two text conditions expressed less hope than those in the control condition. For ***empathy*,** results revealed a significant main effect of text condition, *F*(2, 1074) = 4.65, *p* = 0.009. Individuals in the negative text condition significantly differed in intensity of empathy from the control condition, *t* = –2.56, *p* = 0.01, *d* = 0.18, as did the positive text condition compared to the control condition, *t* = –2.64, *p* < 0.01, *d* = 0.17. No differences were found between the positive text and negative text conditions, *t* = –1.49, *p* > 0.01. Individuals in the control condition expressed significantly more empathy than individuals in the two text conditions.

For ***anger***, results revealed a main effect of text condition, *F*(2, 1074) = 18.96, *p* < 0.001. A significant difference in intensity of anger was found between the negative text condition and the control condition, *t* = –5.61, *p* < 0.001, *d* = 0.44, between the positive text condition and the control condition, *t* = –4.80, *p* < 0.001, *d* = 0.23, and between the positive text condition and negative text condition, *t* = –2.17, *p* = 0.03, *d* = 0.07. Both texts had the effect of reducing individuals’ anger about the pandemic compared to the control condition, but more so in the negative text condition compared to the positive text condition. No differences were found for anxiety between text conditions, *F*(2, 1074) = 2.09, *p* = 0.12.

For ***sadness***, results revealed a significant main effect of text condition, *F*(2, 1074) = 3.78, *p* = 0.02. Individuals in the negative text condition expressed a similar level of sadness compared to the control condition, *t* = 1.86, *p* = 0.24, whereas a significant difference was found between the positive text condition and the control condition, *t* = –2.75, *p* = 0.006, *d* = 0.19 with participants in the control condition reporting higher levels of sadness. A significant difference was also found between the positive text condition and negative text condition, *t* = 2.40, *p* = 0.02, *d* = 0.11, with the positive text condition expressing the least amount of sadness. Finally, for ***hopelessness***, there was a significant main effect of text condition *F*(2, 1074) = 5.08, *p* = 0.006. Specifically, participants in the negative text condition reported significantly higher levels of hopelessness compared to the control group, *t* = 2.17, *p* = 0.03, *d* = 0.15, whereas individuals in the positive text condition reported similar levels of hopelessness compared to the control group, *t* = –1.09, *p* = 0.27. Significant differences were also found between the positive text condition and the negative text condition, *t* = –2.46, *p* = 0.01, *d* = 0.24, wherein participants in the negative text condition reported higher levels of hopelessness.

### Emotions as mediators of text effects

For the last research question, *we examined whether emotions predicted and mediated willingness to engage in prevention action*, and actual preventive action 1 week following intervention. To reduce complexity, we report significant results only. Results revealed that happiness (ß = –0.12, *p* = 0.002), empathy (ß = 0.17, *p* < 0.001), anxiety (ß = 0.13, *p* < 0.001), sadness (ß = 0.10, *p* = 0.01), and hopelessness (ß = –0.13, *p* = 0.001) significantly predicted individuals’ willingness to engage in preventive action. That is, the greater their happiness and sense of hopelessness, the less willing they were to engage in personal preventive action, whereas the higher their levels of empathy, anxiety and sadness, the more willing individuals were to engage in preventive action. Results from mediation analyses further revealed that happiness (indirect effect = 0.03, bootstrap CI from 0.009 to 0.05) and hopelessness (indirect effect = 0.02, bootstrap CI from –0.04 to –0.01) mediated the relationship for differences between the negative text condition and the control condition on willingness to engage in preventive action, whereas sadness (indirect effect = 0.02, bootstrap CI from –0.04 to –0.01) mediated the relationship for differences between the positive text condition and the control condition. Anxiety also mediated the relationship for differences between the positive text condition and negative text condition for willingness to engage in preventive action (indirect effect = –0.01, bootstrap CI from –0.03 to —0.0005).

For willingness to socially distance and isolate, results revealed that happiness (ß = –0.14, *p* < 0.001), empathy (ß = 0.13, *p* < 0.001), anger (ß = –0.11, *p* = 0.001), anxiety (ß = 0.14, *p* < 0.001), and hopelessness (ß = –0.14, *p* = 0.03) were significant predictors. That is, the more empathy and anxiety that individuals experienced, the more willing they were to socially distance and isolate, whereas the more happy, angry, and hopeless they felt, the less willing they were to engage in social distancing and isolation behaviors. Results from mediation analyses further revealed that happiness (indirect effect = 0.04, bootstrap CI from 0.015 to 0.07) mediated the relationship for differences between the negative text condition and the control condition on willingness to socially distance and isolate, whereas empathy (indirect effect = –0.02, bootstrap CI from –0.05 to –0.001; indirect effect = –0.02, bootstrap CI from –0.05 to –0.01) mediated the relationship for differences between the negative text condition and the control condition, as well as between the positive text condition and the control condition. Moreover, anger mediated the relationship for both text conditions (indirect effect = 0.05, bootstrap CI from 0.01 to 0.08 for the negative text; indirect effect = 0.04, bootstrap CI from 0.01 to 0.07 for the positive text).

For actual preventive action at 1 week delay, results revealed that empathy (ß = 0.07, *p* = 0.03) and anxiety (ß = 0.08, *p* = 0.03) were significant predictors, and that empathy mediated the relationship for both text conditions (indirect effect = –0.01, bootstrap CI from –0.03 to –0.006 for the negative text; indirect effect = –0.01, bootstrap CI from –0.01 to –0.001 for the positive text). For social distancing and isolation at 1 week delay, results revealed that happiness (ß = –0.11, *p* = 0.01) and anger (ß = –0.08, *p* = 0.04) were significant negative predictors, whereas anxiety was a significant positive predictor (ß = 0.08, *p* = 0.04). That is, the more happiness and anger individuals experienced, the less likely they were to engage in social isolation or distancing. In contrast, the more anxiety they experienced, the more likely they were to engage in social distancing and isolation. Finally, for risky social behaviors, none of the emotions were significant predictors.

### Supplemental analyses

Given that approximately 36% of our sample was not that concerned about the pandemic, we conducted a supplemental analysis to assess whether level of concern moderated relations between conditions. That is, it could be that individuals who were already concerned about the pandemic (in our sample, approximately 24%) were willing to engage in preventive action and required no persuasion. In contrast, those who were minimally or moderately concerned required a shift in beliefs, and the text conditions may have had variable effects on these individuals. Accordingly, we conducted a moderated mediation using PROCESS (Model 8) to explore the potential effects of level of concern on emotions and willingness and actual preventive action outcomes. Results revealed that level of concern did not moderate relations between text condition on any of the preventive outcomes (*p*-values ranged from 0.14 to 0.99). These results suggest that the texts had similar effects across all levels of concern.

We then examined whether change in emotions from pre-test to post-test was moderated by level of concern across the two text conditions. Indeed, results revealed that individuals who were not that concerned about the pandemic were significantly higher in their pre-test level of happiness than those who were moderately to very concerned, but that level of happiness was significantly lower at post-test for both groups but more so for those who were not that concerned originally, *F*(1, 672) = 10.26, *p* < 0.001, partial *η^2^* = 0.01. Similarly, for sadness, individuals who were not that concerned about the pandemic situation reported lower levels of sadness at pre-test compared to those who were concerned at pre-test, but then reported higher levels of sadness at post-test compared to those who were concerned, *F*(1, 672) = 13.81, *p* < 0.001, partial *η^2^* = 0.013. The same pattern was found for anxiety, *F*(1, 672) = 27.45, *p* < 0.001, partial *η^2^* = 0.03, whereby individuals who were initially not that concerned about the pandemic expressed lower levels of anxiety at pre-test compared to those who were concerned, but then reported higher levels of anxiety at post-test compared to those who were already concerned.

## Discussion

Across four countries at different phases of the COVID-19 pandemic, the efficacy of persuasive texts in increasing individuals’ willingness to engage in preventive action, and in increasing actual preventive action 1 week following the intervention was explored. A second goal was to identify the mechanisms involved in persuading individuals to change their behaviors. Results revealed that individuals who were given the positive persuasive text were more willing to engage in preventive action compared to individuals in the negative text condition and control condition. Results also revealed that individuals who were given the positive persuasive text were more willing to engage in social distancing and isolation than individuals in the control condition. These differences ranged from small to medium in effect size. These results are particularly noteworthy because other studies conducted at the same time showed that interventions were not effective in boosting intentions to engage in social distancing or isolation (Jordan et al., 2020; Pfattheicher et al., 2020). Furthermore, beyond willingness to engage in preventive action, the findings suggest that the effects extended to actual preventive behavior; individuals who received the positive persuasive text reported engaging in fewer risky behaviors (i.e., not engaging in social distancing and isolation) compared to individuals in the control condition. Finally, across all outcomes of interest, no differences were found between individuals in the negative text condition compared to the control condition.

For differences in emotions across conditions, the negative text significantly reduced individuals’ happiness, hope, empathy, and anger about the pandemic situation, whereas the positive text reduced individuals’ hope, anger, empathy, and sadness. These results suggest that the texts had more complex effects on individuals’ emotions than we originally hypothesized. We expected the positive text to maintain or increase positive emotions (i.e., hope, empathy) and decrease negative emotions (i.e., anger, anxiety, sadness, hopelessness), and the negative text to decrease positive emotions (i.e., happiness) and increase negative ones (i.e., anxiety, sadness). Rather, these decreases in both positive and negative emotions had the effect of prompting individuals to take action for those in the positive persuasive text condition. That is, while individuals across all three conditions were primarily willing to take action (and took preventive action), the positive text persuaded individuals significantly more.

From a theoretical standpoint, a decrease in negative emotions should result in greater elaboration and assimilation of the information presented in the text (Pekrun et al., 2009; Muis et al., 2015; Bohn-Gettler, 2019), which we expected would translate into a deeper understanding of the seriousness of the situation and more willingness to engage and actually take preventive action. As Bohn-Gettler (2019) argued, the role that emotions play during text processing depends on the context. In this case, the context was a negative situation that already elicited negative emotions. As such, it appears that in the case of a pandemic situation, reducing negative emotions may have played a more prominent role in social persuasion than maintaining or eliciting more positive emotions (see also Trevors et al., 2021). Under this context, it may have been particularly challenging to elicit more positive emotions and, as such, a reduction in negative emotions was a necessary alternative.

Alternatively, as Bohn-Gettler (2019) argued, a reduction in positive emotions may be necessary to ensure individuals do not ignore belief inconsistent information. For example, for those individuals who did not believe the pandemic to be a serious situation, they expressed higher levels of happiness prior to the presentation of the texts compared to those who were already concerned. Presentation of the texts, particularly for the negative text, had the effect of reducing individuals’ level of happiness, thereby increasing perceptions of the seriousness of the pandemic and willingness to and actually engage in preventive action. Moreover, for those who were not as concerned about the pandemic at pre-test, their levels of anxiety and sadness were initially lower compared to those who were concerned about the pandemic, but then became higher after reading the texts. This suggests that an increase in negative emotions for these individuals resulted in them taking the pandemic situation more seriously, thereby increasing preventive action. Indeed, under this condition, more negative emotions may have resulted in more accommodation of their existing beliefs, which is consistent with Bohn-Gettler’s (2019) PET framework. Accordingly, it appears that emotions played a significant role in persuading individuals to take preventive action.

### Taking preventive action

Both positive and negative texts were designed to increase preventive action and social distancing/isolation by combining personal benefit messages (Jordan et al., 2020) to increase preventive action, and empathic messages (Pfattheicher et al., 2020) to increase social distancing/isolation. The key difference between texts was message framing: positive (saving lives) versus negative (number of deaths). Indeed, the positive text was more effective for increasing willingness and taking action compared to the negative and no text conditions whereas the negative text did not have an effect on willingness or taking action compared to the no text condition. This is consistent with previous research on message framing and behavioral change (Jones et al., 2003), and has important implications for public health messaging. To prompt individuals to take action, it is better to provide persuasive messages in a positive light (i.e., saving lives) rather than a negative one (death).

What was particularly noteworthy was that the positive persuasive text continued to effect actual preventive action 1 week following the intervention and decreased individuals’ risky behaviors with regard to some of the social distancing and isolation behaviors (e.g., not having visitors or face-to-face gatherings, not shopping for non-essential goods). It did not, however, have a significant impact on other social distancing and isolation behaviors (e.g., keeping at least 6 m apart from people who did not live with them). It may be the case that the government actions or lockdowns in place across each of the countries limited individuals’ behaviors and our texts had no effect above and beyond those government actions.

### Potential causal mechanism: Emotions

Collectively, individuals’ emotions about the pandemic situation predicted their willingness to engage in preventive action. The greater individuals’ happiness and hopelessness about the pandemic situation, the less willing they were to engage in preventive action and social distancing/isolation. Happiness can lead to undue optimism and an underestimation of risks (see, e.g., Herrero-Fernández et al., 2020), suggesting that a reduction in happiness can contribute to engaging in preventive behavior. This, however, was not the case for individuals in the negative text condition. Individuals in the negative text condition also reported a significant increase in hopelessness, which may have overshadowed the reduction in happiness whereby they felt that engaging in preventive action would not help. As previous research has shown, hopelessness has detrimental effects on learning and achievement (Burić and Sorić, 2012) and can lead to individuals disengaging altogether (Pekrun and Stephens, 2012).

Individuals who expressed more anger about the pandemic were also less likely to engage in social distancing/isolation. It may be the case that individuals were angry about the lockdowns, about having their freedoms reduced or removed (Jost, 2017). In contrast, the more empathy and anxiety individuals experienced, the more willing they were to engage in preventive action and social distancing/isolation. Moreover, the more sadness individuals experienced, the more willing they were to engage in preventive action. Indeed, these emotions further mediated relations between text conditions and preventive outcomes, which may help to explain why the positive persuasive text had more of an effect on individuals’ willingness and actual preventive action compared to the other two conditions.

For instance, happiness mediated relations between the effects of text condition on both willingness to engage in preventive action and social distancing/isolation. Specifically, the negative text condition significantly reduced individuals’ level of happiness about the pandemic situation, which may have had the effect of reducing processes to assimilate information into existing knowledge structures (Bohn-Gettler, 2019) compared to the positive persuasive text condition for those who already took the pandemic situation seriously. In essence, decreasing these individuals’ positive emotions may have had a backfire effect. The opposite may have occurred for those individuals who did not initially take the pandemic situation seriously. Moreover, for individuals in the negative text condition, their level of hopelessness significantly increased, which also mediated relations between text conditions and outcomes. This increase in negative emotions may have resulted in a significant decrease in processing of the text-based information with regard to what individuals can do to take action. In contrast, for individuals in the positive text condition, their level of happiness was similar to those in the control condition. Their hopelessness did not increase, whereas their level of sadness about the pandemic decreased, as did their anger. Given that these emotions were significant mediators, it may be the case that significantly reducing individuals’ negative emotions benefited them in terms of fostering assimilation of information into existing knowledge structures (Bohn-Gettler, 2019), thereby increasing their willingness to engage in and actually take preventive action for those who were already concerned about the pandemic situation at pre-test.

### Limitations and future directions

Taken together, results from this study suggests that modifying emotions may be critical for persuading individuals to change their behaviors, and that modification may depend on their initial perceptions of the seriousness of the situation. These results support previous theoretical and empirical work, which suggests that higher levels of negative emotions can reduce effortful processing of information given the decrease in attentional resources available (Meinhardt and Pekrun, 2003; Muis et al., 2015), but also support the notion that increasing negative emotions can result in accommodation of current beliefs to allow new incoming information to be integrated into existing structures (Bohn-Gettler, 2019). Reducing negative emotions in the positive persuasive text condition may have been critical to maintain or increase attentional resources so that individuals could process the content more deeply, particularly for those who took the pandemic situation seriously. For individuals in the negative text condition, a decrease in positive emotions and an increase in negative emotions may have led to a decrease in assimilative processing (Bohn-Gettler and McCrudden, 2022). Finally, consistent with Bohn-Gettler and McCrudden (2022), it may also be the case that individuals in the positive persuasive condition, who experienced less negative emotions, spent more time reading belief-inconsistent information particularly for those who initially believed the pandemic was not that serious. This focus may have shifted individuals’ beliefs about the pandemic, resulting in them taking more preventive action. As this is speculative, future research is necessary to better understand precisely how individuals processed information and how emotions played a role. Indeed, one limitation of this research is that we did not measure the cognitive and metacognitive processes individuals used to process the text-based information. Future research is needed to examine how emotions and cognitive and metacognitive processes work to facilitate or constrain behavioral change.

Future research is also needed to assess the mediating role of emotions in inducing positive attitudes toward strategies to reduce the spread of COVID-19 when the content of the message includes both affective appeals and cognitive appeals. Affective appeals focus on the positive or negative feelings or emotions that individuals have toward an attitude object (e.g., taking preventive action may make an individual feel happy about saving lives), whereas cognitive appeals focus on positive or negative attributes about the attitude object (e.g., taking preventive action slows the spread of COVID-19). In the literature on persuasion, research has shown a structural matching effect whereby individuals’ preference for affective information [e.g., high in need for affect (Maio and Esses, 2001)] predicts greater persuasion in response to a message that is affectively based but not cognitively based (Di Plinio et al., 2022), whereas the converse is true when individuals’ preference for information is cognitively based (e.g., high need for cognition; Cacioppo and Petty, 1982).

Recently, Giammusso et al. (2022) explored how matched (same valence) or mixed (different valence) messages that included both affective and cognitive appeals changed attitudes about COVID-19 preventive action. They found that individuals who were high on need for affect but low on need for cognition changed their attitudes according to the affective appeal of the message (e.g., negative affective message resulted in more negative attitudes; positive affective messages resulted in more positive attitudes), regardless of the valence of the cognitive content. In contrast, individuals who were high on need for cognition but low on need for affect were not affected by cognitive or affective appeals, regardless of the valence of those appeals. How emotions might directly mediate or moderate this effect should be explored.

A second limitation is that the texts were not identical in length. The positive text was slightly longer, and the longer text may have been more persuasive due to simple length rather than content. Future work is needed to rule out this possibility. Additionally, individuals reported what they thought they did over the preceding week and were not specifically asked to keep track of their preventive actions. Future research should use alternative methods like diaries to provide a more accurate picture of actual behavior. Fourth, the follow-up was only 1 week following the intervention. It is not possible to assess whether the messages continued to have an impact for a longer period of time, or whether the messages interacted with governmental actions taken as the pandemic pressed on. We also used convenience sampling, which limits generalizability of the findings. Finally, we did not include a baseline measure of what individuals were already doing to take preventive action, which would have provided a more in-depth analysis as to the kind of impact our messages had on preventive action above and beyond what individuals were already doing. Future research should also undertake a more in-depth approach (e.g., *via* interviews or other qualitative methods) with individuals to better understand what other mechanisms are at play when it comes to social persuasion. It may be the case that concurrent governmental actions interact with public messages and personal or societal values (e.g., Trevors, 2022) to influence intentions and actual behavior. Finally, it is important to note that more than half of our sample was politically liberal, which may have reflected a biased sample. Future work is needed with more individuals from multiple political camps.

## Conclusion

Taken together, this research shows that persuasive messages can influence both people’s willingness to engage in preventive actions suited to reduce the spread of COVID-19, and their actual preventive behavior as well as reduction of risky behavior. However, these effects are not easy to achieve, and may depend on message tailoring or targeting, as explained by the ELM (Petty and Cacioppo, 1986). Tailoring is defined as using any combination of information or behavior change strategies that is best suited to reach specific persons based on characteristics that are unique to those individuals, and derived from prior assessment (Kreuter et al., 2000). Targeting involves aiming messages at particular groups of people based on identifiable characteristics (e.g., political ideology; Butterfuss et al., 2020), such as emotional profile in the case of COVID-19. Indeed, research has shown that matching health messages to personal characteristics can increase the effectiveness of the message in changing behaviors (see Kroeze et al., 2006, for a review). As such, future studies should examine if the effectiveness of persuasive messages in changing pandemic-related behaviors can be further boosted by first assessing individuals’ emotional profile within a given socio-cultural and historical context, and then tailoring the message accordingly. We argue that it is just as important to conduct a linguistic analysis on any future texts used to ensure they are of the correct valence. These results also have important policy implications for educationally based interventions used by governments in terms of tailoring messages during a pandemic crisis.

## Data availability statement

The raw data supporting the conclusions of this article will be made available by the authors, without undue reservation.

## Ethics statement

The studies involving human participants were reviewed and approved by the McGill University, University of Southern California, University of Essex, University of Munich, University of Minnesota, University of Padova. The patients/participants provided their written informed consent to participate in this study.

## Author contributions

KM, GS, RP, PK, and LM designed the study. KM collected the data in Canada, analyzed the data across countries, and wrote the manuscript. All authors collected data in their respective countries, analyzed data, and wrote portions of the manuscript.
