# Corrigendum: Flattening the COVID-19 curve: Emotions mediate the effects of a persuasive message on preventive action

**DOI:** 10.3389/fpsyg.2023.1148252

**Published:** 2023-02-02

**Authors:** Krista Renee Muis, Gale M. Sinatra, Reinhard Pekrun, Panayiota Kendeou, Lucia Mason, Neil G. Jacobson, Wijnand Adriaan Pieter Van Tilburg, Ellen Orcutt, Sonia Zaccoletti, Kelsey M. Losenno

**Affiliations:** ^1^Department of Educational and Counselling Psychology, Faculty of Education, McGill University, Montreal, QC, Canada; ^2^Rossier School of Education, University of Southern California, Los Angeles, CA, United States; ^3^Department of Psychology, University of Essex, Colchester, United Kingdom; ^4^Department of Educational Psychology, College of Education and Human Development, University of Minnesota Twin Cities, St. Paul, MN, United States; ^5^Department of Developmental Psychology and Socialisation, University of Padua, Padua, Italy

**Keywords:** social persuasion, intervention, emotions, COVID-19, cross-cultural research

In the published article, an author name was incorrectly written as Neil Jacobs. The correct spelling is Neil G. Jacobson.

The authors apologize for this error and state that this does not change the scientific conclusions of the article in any way. The original article has been updated.

