# Supplementary material for: Flattening the COVID-19 curve: Emotions mediate the effects of a persuasive message on preventive action
Source: Front Psychol. 2022 Dec 1;13:1047241. doi: 10.3389/fpsyg.2022.1047241 (PMC9751357; doi:10.3389/fpsyg.2022.1047241)

**Appendix**

***Negative Text***

Please read the following information about COVID-19, which the World Health Organization has recently classified as a pandemic.

Coronavirus disease 2019 (COVID-19) is a respiratory illness that can spread from person to person. The virus that causes COVID-19 is a novel coronavirus that was first identified during an investigation into an outbreak in Wuhan, China. Because COVID-19 is a novel virus, there is no immunity in the community yet. There is also no vaccine for COVID-19.

COVID-19 is currently spreading rapidly throughout Canada and other parts of the world. As of April 17, there are at least 30,106 confirmed cases in Canada, and 2,173,432 world-wide. These numbers are likely a major underestimate given that not everyone is being tested. In many places around the world, the number of cases is growing exponentially. According to the Public Health Agency of Canada, between 1.9 to 18.5 million people in Canada could be infected over the course of the pandemic, and as many as 11,000 to 350,000 people could die. The calculations based on these scenarios suggested 146,000 to 1.4 million people in Canada could require hospitalization, potentially crushing the nation’s medical system, which has about 57,000 staffed hospital beds. As of today, Canada has only 3,170 ICU beds and 4,982 ventilators.

COVID-19 is very contagious. And, people can spread COVID-19 before experiencing any symptoms.

This means coronavirus is a serious threat to you and your community. It is recommended that you take this threat very seriously to prevent contracting COVID-19 and getting very ill or dying or spreading COVID-19 and causing people in your community to get very ill or die.

Fortunately, there are steps you can take to keep yourself and your community safe.

It is recommended that you practice good personal hygiene (wash your hands, avoid shaking hands or hugging others, avoid touching your face, and cover your mouth when you cough or sneeze), stay home if you are even a little bit sick, and practice social/physical distancing (staying at least two to three metres [6 to 10 feet] away from others).


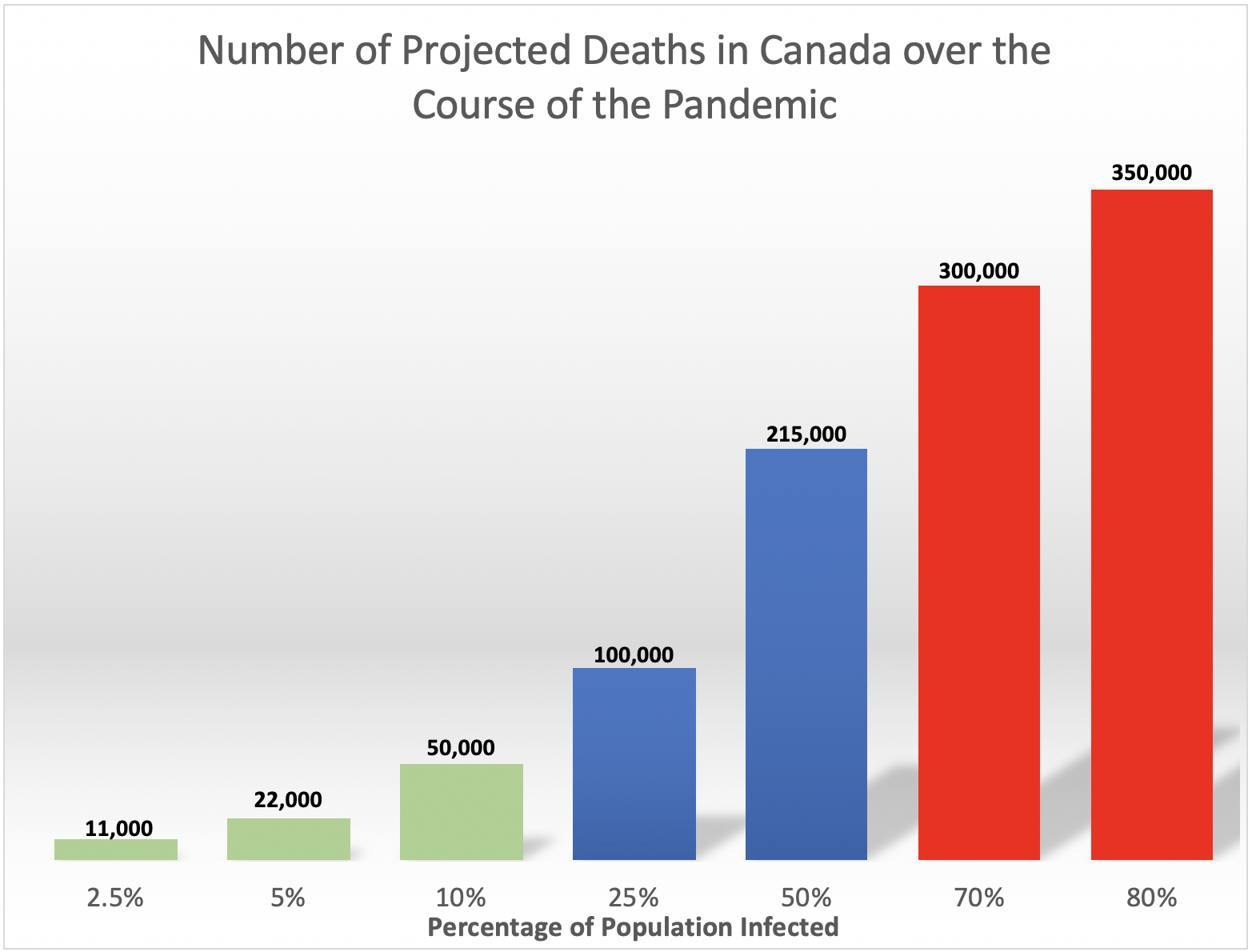


***Positive Text***

Please read the following information about COVID-19, which the World Health Organization has recently classified as a pandemic.

Coronavirus disease 2019 (COVID-19) is a respiratory illness that can spread from person to person. The virus that causes COVID-19 is a novel coronavirus that was first identified during an investigation into an outbreak in Wuhan, China. Because COVID-19 is a novel virus, there is no immunity in the community yet. There is also no vaccine for COVID-19.

COVID-19 is currently spreading rapidly throughout Canada and other parts of the world. As of April 17, there are at least 30,106 confirmed cases in Canada, and 2,173,432 world-wide. These numbers are likely a major underestimate given that not everyone is being tested. In many places around the world, the number of cases is growing exponentially. According to the Public Health Agency of Canada, between 1.9 to 18.5 million people in Canada could be infected over the course of the pandemic, and as many as 11,000 to 350,000 people could die. The calculations based on these scenarios suggested 146,000 to 1.4 million people in Canada could require hospitalization, potentially crushing the nation’s medical system, which has about 57,000 staffed hospital beds. As of today, Canada has only 3,170 ICU beds and 4,982 ventilators.

This means coronavirus is a serious threat to you and your community. It is recommended that you take this threat very seriously to prevent contracting COVID-19 and getting very ill or dying or spreading COVID-19 and causing people in your community to get very ill or die.

Provincial governments across Canada have taken action to intervene and stop the spread of COVID-19 by closing schools, businesses, and other non-essential services. Canada has closed its borders to the US, and travel between provinces is no longer allowed unless deemed essential. Although provinces are doing what they can do stop the spread, the Public Health Agency of Canada says it is not enough.

Fortunately, there are important preventive actions you can take to keep yourself and your community safe; actions that will save thousands of lives. The World Health Organization (WHO) and Centers for Disease Control and Prevention (CDC) have recommended that you practice good personal hygiene (wash your hands with soap for 20 seconds, avoid shaking hands or hugging others, avoid touching your face, and cover your mouth when you cough or sneeze), stay home if you are even a little bit sick, and practice social/physical distancing (staying at least two to three metres (6 to 10 feet] away from others).

The figure below shows different scenarios in terms of people taking preventive actions and number of projected deaths in Canada. If *everyone* takes preventive action now, we can limit the spread of infections to 2.5% to 10% of the population and save 300,000 to 339,000 lives. However, if only *some people* take preventive action, then 25% to 50% of the population will contract the virus and as many as 215,000 will die. The red bars show what would have happened if we had done nothing. If we did nothing, 70% to 80% of the population would contract the virus and 350,000 people in Canada would die.

The more preventive action we take, the more lives we will save. Please save lives. Do your part in taking preventive action now.

1. COVID-19 in Canada: Using data and modeling to inform public health action: Technical briefing for Canadians. Public Health Agency of Canada. April 9, 2020.


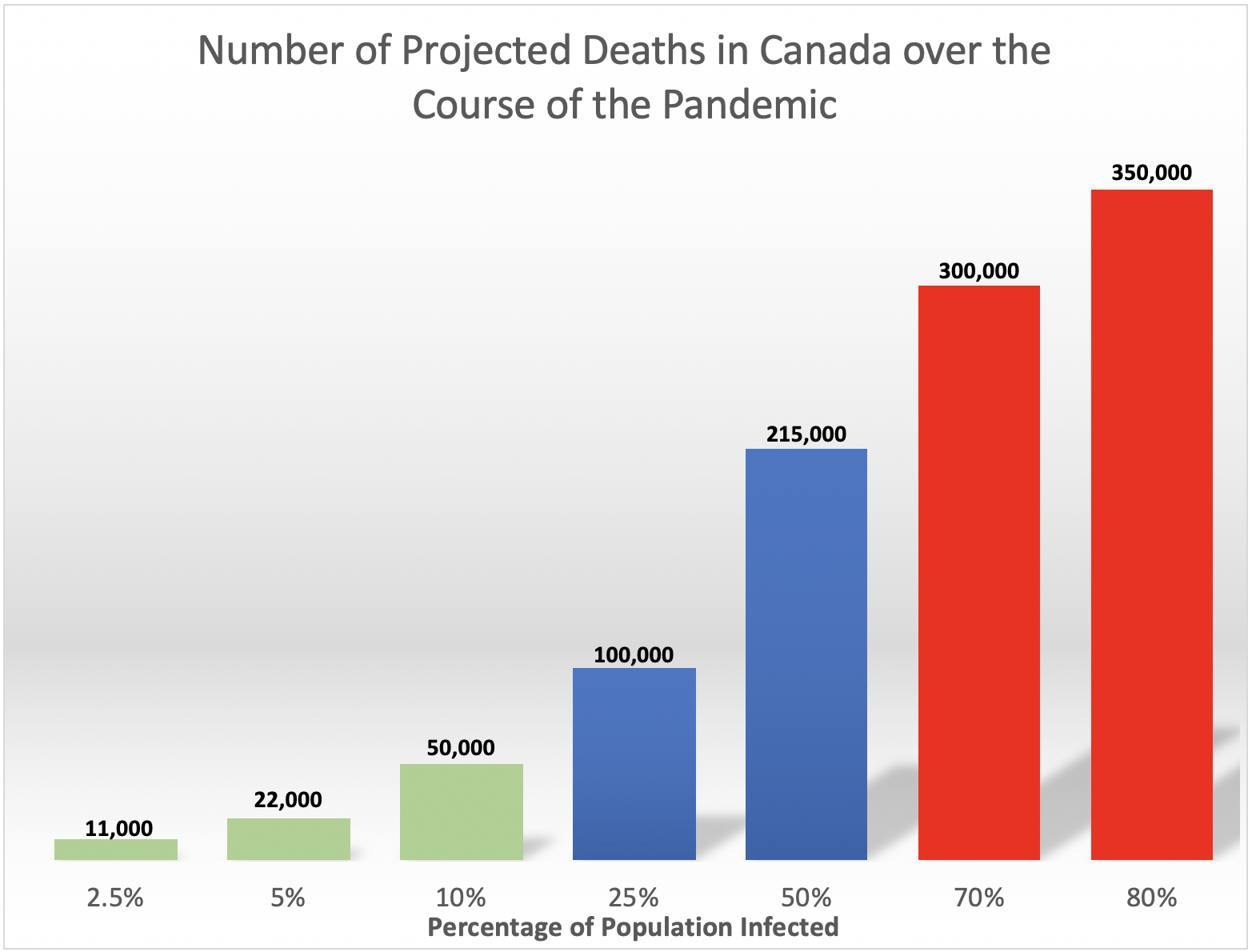

Supplement: Supplementary file 1 [file Data_Sheet_1.docx]
